# Identification of Illicit Conservation Treatments in Fresh Fish by Micro-Raman Spectroscopy and Chemometric Methods

**DOI:** 10.3390/foods12030449

**Published:** 2023-01-18

**Authors:** Elisa Robotti, Masho Hilawie Belay, Elisa Calà, Alessandro Benedetto, Simone Cerruti, Marzia Pezzolato, Francesco Pennisi, Maria Cesarina Abete, Emilio Marengo, Paola Brizio

**Affiliations:** 1 Department of Sciences and Technological Innovation, University of Piemonte Orientale, Viale Michel 11, 15121 Alessandria, Italy; 2 Istituto Zooprofilattico Sperimentale del Piemonte, Liguria e Valle d’Aosta, Via Bologna 148, 10154 Torino, Italy

**Keywords:** Cafodos-like treatment, chemometrics, fresh sea bass, food preservation, micro-Raman, muscle and skin, PLS-DA

## Abstract

In the field of food control for fresh products, the identification of foods subjected to illicit conservation treatments to extend their shelf life is fundamental. Fresh fish products are particularly subjected to this type of fraud due to their high commercial value and the fact that they often have to be transported over a long distance, keeping their organoleptic characteristics unaltered. Treatments of this type involve, e.g., the bleaching of the meat and/or the momentary abatement of the microbial load, while the degradation process continues. It is therefore important to find rapid methods that allow the identification of illicit treatments. The study presented here was performed on 24 sea bass samples divided into four groups: 12 controls (stored on ice in the fridge for 3 or 24 h), and 12 treated with a Cafodos-like solution for 3 or 24 h. Muscle and skin samples were then characterized using micro-Raman spectroscopy. The data were pre-processed by smoothing and taking the first derivative and then PLS-DA models were built to identify short- and long- term effects on the fish’s muscle and skin. All the models provided the perfect classification of the samples both in fitting and cross-validation and an analysis of the bands responsible for the effects was also reported. To the best of the authors’ knowledge, this is the first time Raman spectroscopy has been applied for the identification of a Cafodos-like illicit treatment, focusing on both fish muscle and skin evaluation. The procedure could pave the way for a future application directly on the market through the use of a portable device.

## 1. Introduction

In recent years, fish and global seafood consumption have strongly increased worldwide [1,2]. Simultaneously, consumers’ demand for food safety has arisen and the topic of seafood authenticity has become of pivotal importance [3]. Indeed, seafood frauds are on the rise, and the Rapid Alert System for Food and Feed (RASFF) often states cases of dilution, illicit treatments, and mislabeling, throughout the European Union [4,5,6]. Since one of the main issues about fish and seafood is their quick perishability, it is imperative to consume these kinds of products fresh or properly processed (i.e., frozen, salted, dried, partially cooked, etc.). Moreover, many producers choose to add food additives, such as phosphates, sulfites, and citrate, to preserve seafood from natural chemical and biological alteration [7,8,9,10]. From this perspective, it is documented that criminals and gray market vendors adulterate seafood by adding unknown or undeclared (often illegal) substances, in order to enhance freshness or other quality attributes [11,12]. The main rationale for such fraud is economical gain; indeed, the American Food and Drug Administration (FDA) coined the term “economically motivated adulteration” (EMA) in 2009 to describe “the fraudulent, intentional substitution or addition of a substance in a product for the purpose of increasing the apparent value of the product or reducing the cost of its production” [13]. For this reason, it is still necessary to rely on accurate and trustworthy methods to aid official controls and even suppliers in detecting such malpractices. To date, plenty of techniques have been described to uncover illicit treatments and adulteration in seafood, such as histology, ion chromatography, protein analysis, determination of biogenic amines, etc. [14,15,16,17]. In particular, the detection of biogenic amines, such as histamine, is historically used to assess the freshness in seafood: they can be produced during degradation triggered by bacteria and specific spoilage organisms (SSO) and, if present in high amount, may cause a disease known as “scombroid food poisoning” in consumers [18].

It is, therefore, easy to understand why an additive that hides the signs of spoilage in fish can become very dangerous for consumers, and particularly for chronically allergic people. Here, we will focus on an adulterant characterized by this property and known in Italy as “Cafodos”: it is a mixture of citric acid, sodium citrate and hydrogen peroxide (the latter is prohibited in fish products [19]), added to restore some shine to the fish skin and prolong its shelf-life through a bactericidal effect [7,8,20,21,22]. Furthermore, it has recently become famous, because of its property to “disappear” under major control techniques, due to its volatility and rapid assimilation of its ingredients [7,8,20,21]. Its use results in an improved appearance especially of fish filets and an increase in retained water [23,24] when it is coupled to polyphosphates, thus, making old and altered products look fresher and juicier. The main consequence is the economic profit for sellers, who earn from rotten products which should be excluded from trade. Actually, the “Cafodos” mixture appears to be non-toxic to humans at low concentrations, but the greatest risks to human health are connected to the direct ingestion of spoiled fresh-looking fish.

It is, therefore, important to develop procedures able to detect this type of treatment. Current technologies are able to detect citric acid and its salts, but not hydrogen peroxide, as well as the presence of polyphosphates. From this perspective, spectroscopic techniques based on the infrared and near-infrared (780–2500 nm) regions can offer a valuable contribution for evaluating the biochemical properties of tissues, such as protein and fat content, water-binding, total volatile basic nitrogen, trimethylamine and other amines [25]. Some studies have also successfully detected the presence of hydrogen peroxide in fish and cuttlefish [26,27]. 

In general, spectroscopic techniques have the key advantage of requiring a smaller quantity of reagents, or no reagents at all, compared to more traditional analytical methods, as biogenic amines determination by HPLC-UV [28]), volatile amines detection by GC-MS [29]) and lipid derivatives determination [30]. 

NIR and Raman spectroscopies have already been used in seafood analysis, coupled with multivariate analysis, to, e.g., discriminate fresh from frozen/thawed products and wild-caught from farmed fish, identify the taxonomic species, and assess the freshness of products [31,32,33,34,35,36,37,38,39,40,41]. Raman spectroscopy, in particular, provides information about the secondary and tertiary structure of proteins [42,43] and results from this technique have already been compared with the results of methods commonly used to evaluate the physico-chemical properties of proteins and chemical parameters such as the dimethylamine content [44], in fish meat samples stored at different temperatures and for different periods of time. Moreover, Raman spectroscopy can provide information about the modifications in lipids, which are ultimately due to the oxidation and hydrolysis of fatty acids [45,46,47]. Finally, it can be exploited for classification purposes [48,49,50,51,52,53]. Furthermore, portable devices are very handy and can be applied directly on-site or in the production chain. 

Besides vibrational spectroscopy, nuclear magnetic resonance (NMR) spectroscopy has recently been used to determine the quality and freshness of fish products [54,55,56,57,58,59]; however, when compared to vibrational spectroscopy, NMR has the disadvantage of being more expensive and not portable. 

In this panorama, the present work aimed to use micro-Raman technology to analyze whole fish treated with a Cafodos-like mixture in order to develop innovative methods to detect illicit treatments and adulteration in seafood. Multivariate data analysis was applied to classify treated vs. non-treated fish samples, and multivariate models were built to identify the effect of both short- and long-term exposure to the treatment. The results were then evaluated and discussed.

## 2. Materials and Methods

### 2.1. Materials and Reagents

Hydrogen peroxide solution (≥30%, for trace analysis) and sodium citrate tribasic dihydrate (≥99.0%) were purchased from Merck Life Sciences (Milan, Italy). A Cafodos-like treatment solution (TS) composed of 8 g/L hydrogen peroxide and 2.5% (*w*/*v*) sodium citrate was prepared in ultrapure water and stored in a refrigerator at 4 °C. Fish samples were purchased from a local supplier chain. 

### 2.2. Study Design and Sample Preparation

**Figure 1:**
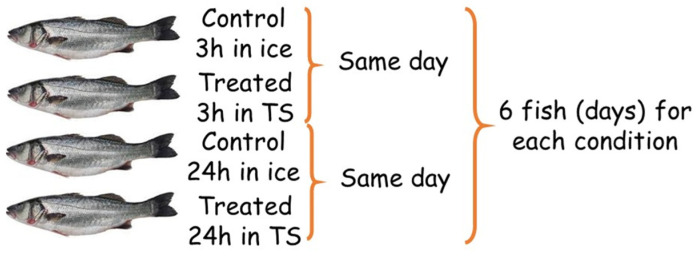
Study design adopted: paired comparisons were carried out. Two fish samples were analyzed each day: one treated and one control fish sample.

The study was performed on European bass (*Dicentrarchus labrax*) samples. The individuals were obtained from a single farm, soon after being caught, through a local supplier chain able to deliver collected animals to retailers in less than 24 h. They were, therefore, homogeneous for production cycle and commercial size (average gutted weight of 600 g). The samples were divided into 4 groups: controls (stored on ice in the fridge for 3 h and for 24 h, respectively) and treated with a Cafodos-like solution (TS) of hydrogen peroxide (8 g/L) and citric acid (2.5%, *w*/*v*) for 3 h and 24 h. The study design reported in Figure 1 was adopted to simultaneously: (i) evaluate the natural variability (expected to be high for the use of animal specimens); (ii) keep as low as possible the number of replications to minimize the experimental effort; (iii) collect the measurements in a short time to provide an evaluation of the fish soon after treatment. The experimentation was carried out over 12 different days, with two samples evaluated and treated as paired comparisons each day: one control and one treated fish. Each of the 4 conditions reported in Figure 1 was therefore evaluated by 6 fish samples, for a total of 24 samples. 

For the treatment, each fish sample was placed in the treating solution (TS) for 60 s, transferred into a food bag and stored on ice in the fridge for 3 h or 24 h. The ratio of fish to TS was 1:1 (*w*/*v*). The corresponding control samples were treated in the same way, by placing the fish sample in ultrapure water. 

After the treatment, each fish was washed by flushing it with deionized water for approximately 2 min and the water was drained. Then, skin and muscle samples for micro-Raman measurements were taken from three different positions of the fish’s middle back (muscle samples were taken from positions close to the removed skin). Each sampled portion of muscle and skin was arranged between two microscope slides for micro-Raman measurements.

A 9-digits label was used to describe each measurement: a letter identifying the type of matrix (M = muscle; S = skin); “F” followed by two numbers indicating the number of the fish sample; a letter indicating control (C) or treated (T) samples; two numbers indicating the period on ice-storage in the fridge (03 = 3 h; 24 = 24 h); “_” followed by a progressive number (1, 2 or 3) indicating the replication.

### 2.3. Micro-Raman Spectroscopy

Raman spectra were collected with a high-resolution dispersive Horiba (Villeneuve d’Ascq, France) LabRAM HR Evolution model spectrophotometer coupled to a confocal microscope. The instrument was equipped with a 633 nm excitation red laser, two (600 and 1800 lines/mm) dispersive gratings, an 800 mm path monochromator and a Peltier cooled CCD detector. The optical arrangement gave a spectral resolution of about 2 cm^−1^. Spectra were obtained by placing the samples on the microscope stage and observing them with long working distance 50X objectives. The sampled area was identified and focused using a video camera over the microscope binoculars. Laser power at the sample was kept low by means of neutral density filters, in order to prevent any thermal degradation of the surface molecules, then gradually increased up to the optimal signal-to-noise ratio, which is 50% laser power. Exposure time was 15 s according to needs (3 accumulations), in the spectral range 200–3000 cm^−1^. The system was managed with LabSpec 6 software (Horiba, Villeneuve d’Ascq, France) running under Windows 10™. 

A preliminary evaluation of the sample variability was undertaken: one fish sample was evaluated by 5 replications of a Raman spectrum collected in very close sampling points on both muscle and skin for an evaluation of the repeatability of the measurement. Since the repeatability proved to be high, it was possible to reduce the number of spectral replications and provide the characterization of each sample by collecting three spectra in different positions of each fish for both the skin and the muscle. 

### 2.4. Data Analysis

Smoothing by Savitzky–Golay with step 30 was applied to all spectra, followed by: (1) First derivative by Savitzky–Golay, first order of the polynomial, step 101; (2) Second derivative by Savitzky–Golay, first order of the polynomial, step 101; (3) Standard normal variate (SNV); (4) treatment as in (1) followed by SNV; (5) treatment as in (2) followed by SNV. The best results were obtained by (1), therefore only the results obtained on first derivative spectra have been presented here and briefly compared to the results obtained on raw spectra after baseline correction. 

Since a control and a treated fish were analyzed for each day, the first derivative spectra were mean centered with respect to each analysis day.

#### 2.4.1. Principal Component Analysis (PCA)

PCA [60] is a pattern recognition method that provides a new set of orthogonal variables called principal components (PC), linear combinations of the original variables. PCA provides the scores, which are the projections of the samples on the PCs, and the loadings, which are the coefficients of each variable in the linear combination describing each PC. Scores and loadings are usually analyzed graphically by representing them on the space given by two PCs at a time: the score plot allows the identification of groups of samples with a similar behavior, while the loading plot provides information on the correlations between the variables and about the reasons for the observed sample grouping. PCA was first applied to the overall dataset to provide a global overview of the correlation structure.

#### 2.4.2. Partial Least Squares Discriminant Analysis (PLS-DA)

Partial least squares (PLS) [60] is a multivariate regression method that correlates the system descriptors (X variables) to one or more experimental responses (Y variables). The method searches for pairs of latent variables (LVs), similar to PCs) on both X and Y spaces that mostly correlate. PLS-DA is a modification of PLS for classification purposes [60], where a Y variable is built to represent class membership: −1 is attributed to the control samples, while +1 is given to the other class. Here, PLS-DA was applied to identify:

(1) Short-term effects of the treatment on muscle: the dataset consisted in micro-Raman spectra collected on muscle from six control fish and six 3 h-treated fish (6 fish × 3 replicates = 18 control measurements; 6 fish × 3 replicates = 18 treated measurements).
(2) Long-term effects of the treatment on muscle: the dataset consisted of micro-Raman spectra collected on muscle from six control fish and six 24 h-treated fish (6 fish × 3 replicates = 18 control measurements; 6 fish × 3 replicates = 18 treated measurements).
(3) Short-term effects of the treatment on skin: the same as in (1) but spectra were collected on the skin samples;
(4) Long-term effects of the treatment on skin: the same as in (2) but spectra were collected on the skin samples.

PLS-DA was applied to autoscaled data. Classification was coupled to variable selection in backward elimination: at each iteration, the variables with the smallest VIP score [60], calculated in cross-validation, were eliminated. At each cycle no more than 6% of the remaining variables were eliminated at a time. Cross-validation was applied with a leave-more-out strategy, taking out at each time all the replications of the measurements of the fish samples that were analyzed in the same day (6 cancellation groups).

The classification results were evaluated on the basis of parameters related to the overall classification performances (%Accuracy and Non-Error-Rate—NER%) and to single class results (sensitivity, specificity and precision).

### 2.5. Software

Micro-Raman spectra were recorded by LabSpec 6 software (Horiba, Villeneuve d’Ascq, France). Spectra pre-treatment was carried out through home-made routines developed in Matlab (R2014, The Mathworks, Natick, MA, USA). PLS-DA models were calculated by the Classification Toolbox [61] (Milano Chemometrics Group, Milan, Italy), developed in Matlab. All graphical representations were carried out by Statistica v.7 (Statsoft Inc., Tulsa, OK, USA) and Origin (OriginLab, Northampton, MA, USA). 

## 3. Results

### 3.1. Micro-Raman Spectroscopy

**Figure 2:**
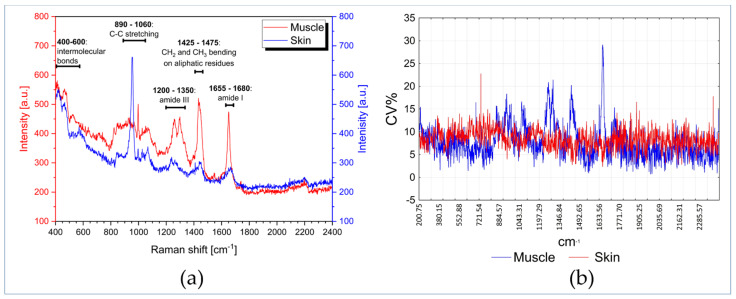
Raman spectra of fish muscle (red) and skin (blue) (**a**); *CV*% for each Raman shift calculated for 5 spectral replications on muscle (red) and skin (blue) (**b**).

Figure 2a shows two examples of Raman spectra, between 400 and 2400 cm^−1^, acquired, respectively, on fish muscle (red) and skin (blue). In the muscle’s spectrum, the most relevant bands are in the region 1200–1700 cm^−1^ and, as reported in the literature [42,62,63], these signals are useful to estimate the secondary structure of proteins. The sharp band at about 1655 cm^−1^ is the amide I band, which can be correlated with proteins having a high α-helix content. At 1660–1680 cm^−1^, another weak and relatively wide amide I band appears as a shoulder of the previous signal, which can be attributed to proteins rich in β-sheet or random-coils structures. On the other hand, the region between 1200 and 1350 cm^−1^ represents the amide III band. However, it is difficult to interpret due to the overlap of signals from proteins high in β-sheet content and those with a higher degree of random-coil structures. Other bands correlated with the secondary structure of proteins are the C-C bond stretching vibrations in the range 890–1060 cm^−1^; interpretation is difficult in this case as well because signals from α-helices (890–945 cm^−1^) and β-sheets (1020–1060 cm^−1^) are wide and not very intense and noise is present. Another sharp band is positioned at ca. 1450 cm^−1^, due to -CH_2_ and -CH_3_ bending vibrations of aliphatic hydrophobic protein residues; this signal can, therefore, be linked to the tertiary structure of proteins, together with other signals from tryptophan and tyrosine [42] which, however, are not clearly visible in the spectrum, probably because they are covered by other bands. Finally, signals from fats can be found in the region between 1260 and 1760 cm^−1^ [45,47]: in particular, signals from unsaturated fatty acids can appear in the same position as the amide I band (ca. 1670–1680 cm^−1^ for the trans configuration and ca. 1650–1665 cm^−1^ for the cis configuration). However, the muscular tissue predominantly consists of proteins; therefore, the first interpretation is more realistic. 

In the skin spectrum, the previously described sharp signals, in the region 1200–1700 cm^−1^ are barely visible; this can be due to a lower content of proteins with α-helix structure. Instead, in the region of C-C stretching vibrations, a strong and narrow signal at ca. 950 cm^−1^ is present. Finally, in both spectra, there are some weak bands in the region below 600 cm^−1^, which are due to intermolecular bonds (hydrogen bonds) between proteins, or even with water molecules [64,65].

As already pointed out in Section 2.3, a preliminary evaluation of the spectral variability was performed. Figure 2b reports the coefficient of variation % (*CV*%) of five replications of Raman spectra collected at very close points on the same fish sample, for both muscle and skin. The *CV*% is reported for each Raman shift and was calculated as:

(1) $$CV{\%}_{j}=\frac{s_{j}}{{\bar{x}}_{j}}*100$$

where: ${\bar{x}}_{j}$ is the average intensity of the *j*-th Raman shift and $s_{j}$ is the standard deviation of the *j*-th Raman shift.

Figure 2b shows that the *CV*% for both skin and muscle is almost always < 15% and in many cases < 10%, proving therefore the good repeatability when measurements are taken close to one another. Due to the good repeatability, each sample (control and treated samples) was therefore evaluated by three measurements taken in three different positions without replications, to obtain a good compromise between sample characterization, experimental effort and time reduction. 

### 3.2. PCA on the Overall Dataset

**Figure 3:**
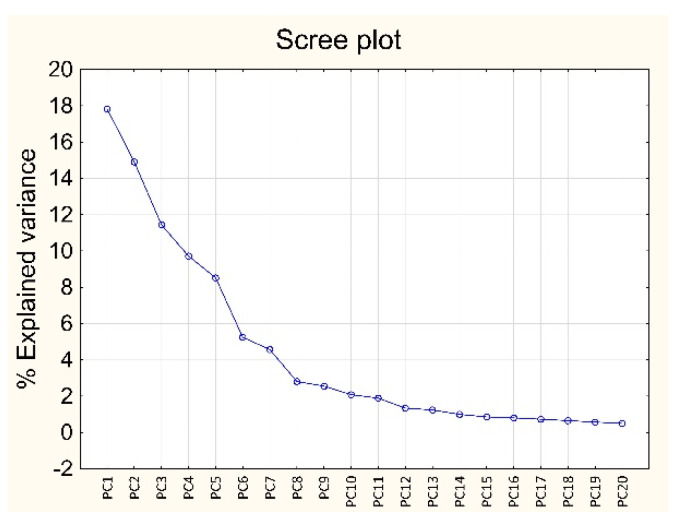
Results of PCA on the overall dataset in first derivative: scree plot.

**Figure 4:**
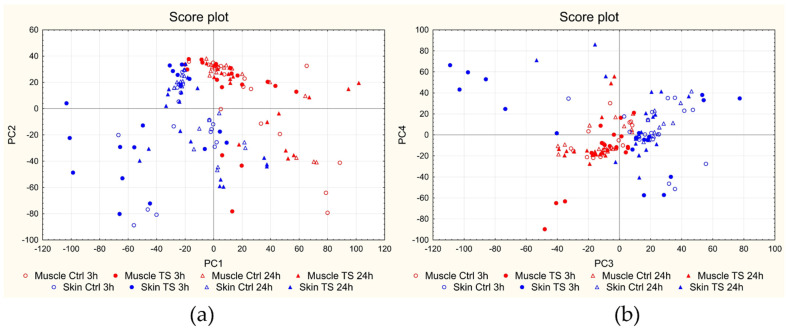
Results of PCA on the overall dataset in first derivative: score plots of PC_2_ vs. PC_1_ (**a**) and of PC_4_ vs. PC_3_ (**b**). Muscle measurements are indicated in red and skin measurements in blue; empty circles = controls at 3 h (18 muscle and 18 skin samples); full circles = 3 h treated (18 muscle and 18 skin samples); empty triangles = controls at 24 h (18 muscle and 18 skin samples); full triangles = 24 h treated (18 muscle and 18 skin samples).

PCA was carried out on the overall dataset described by 144 samples (24 fish samples × 3 replications × 2 matrices) and 7377 variables (Raman shift; Raman spectra were turned into first derivative after smoothing), after autoscaling. Figure 3 reports the scree plot, i.e., the plot of the % of variance (y-axis) explained by each PC (x-axis): the first 4 PCs explain about 54% of the overall information (PC_1_ = 17.82%, PC_2_ = 14.91%, PC_3_ = 11.44%, and PC_4_ = 9.72%). Figure 4a,b reports the score plots of the first four PCs. 

The score plots of the first four PCs show a clear separation of the samples according to the matrix (muscle and skin measurements appear well separated above all on PC_1_ and PC_3_). A certain separation can also be seen between controls and the corresponding treated samples; however, the samples in the score plots are grouped according to the day of analysis in several cases. For this reason, further investigations were carried out on the data centered on the day of analysis.

### 3.3. Classification Models

For each of the four comparisons described in Section 2.4.2, PLS-DA was applied after centering based on each day of measurement and autoscaling. The Y variable was coded so that −1 was attributed to control measurements and +1 to treated samples measurements. PLS-DA was coupled to variable selection in backward elimination: the variables with the smallest VIP score [60], in cross-validation, were eliminated (no more than 6% of the variables simultaneously) at each cycle. The application of cross-validation with ratios between training and test samples of about 80:20 (corresponding to five cancellation groups) is considered common practice in chemometrics; however, in this case, a different ratio was applied to take into consideration the study design, based on paired comparisons carried out in the same day, and obtain a more reliable evaluation of the prediction ability of the models. Cross-validation was therefore applied with a leave-more-out strategy, taking out at each time all the replications of the measurements of the fish samples that were analyzed in the same day (six cancellation groups). For each of the four comparisons, a classification model was calculated, and the corresponding score and coefficients plots are presented here. In the score plots, each measurement is represented in the space given by the first one or two latent variables (LVs) calculated; controls are reported as blue circles and treated samples as red ones. The plots of the coefficients instead report on the x-axis the variables included in each model by the variable selection procedure and on the y-axis the corresponding coefficient for each final model. In the coefficients plots, positive variables correspond to variables with a higher signal in the first derivative of treated samples, while negative coefficients correspond to variables with a lower signal in the first derivative of treated samples.

Table 1 reports the performances of the obtained models comparing the results on raw spectra after baseline correction and on first derivative spectra. The results obtained on the raw data provide a worse classification performance, both in fitting and cross-validation for the short-term treatment on muscle. For the long-term treatment on muscle and both short- and long-term treatments on skin, instead, the classification of the samples was perfect both in fitting and cross-validation considering raw data and first derivative transformed spectra. However, a higher number of variables and LVs were included in the final models in the case of raw spectra: this behavior can be related to a possible overfitting effect. For this reason, only the results obtained for the first derivative transformed spectra are discussed here.

#### 3.3.1. Muscle—Short-Term and Long-Term Treatment Effects

**Figure 5:**
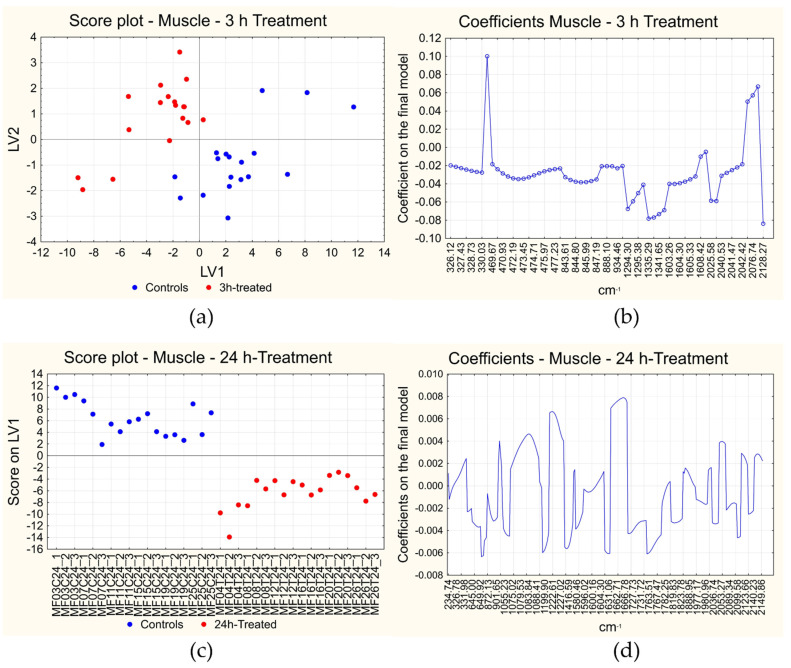
Results of PLS-DA on muscle. Score plot (**a**) and plot of the coefficients (**b**) of short-term treatment and long-term treatment (**c**,**d**). The short-term score plot reports the scores of each measurement in the space given by the first two latent variables (LVs) calculated; the long-term score plot reports the score of each measurement on the first LV (LV_1_) on the y-axis and the measurements on the x-axis. In the score plots the samples are indicated in blue if they are controls and in red if they are treated samples. The plots of the coefficients report the variables included in the final models on the x-axis and the coefficients on the final model on the y-axis. Positive coefficients correspond to variables with a higher signal in the first derivative of treated samples, while negative coefficients correspond to variables with a lower signal in the same situation.

In the case of short-term treatment, the PLS-DA algorithm with variable selection allowed us to obtain a final model with 61 variables and 5 LVs, reaching the perfect classification of all the samples both in cross-validation and in fitting (Table 1), explaining about 74.8% of the information contained in the X variables and the 48.6% of the class belonging (Y variable). The score plot of the first two LVs is reported in Figure 5a, where the two classes of samples appear well separated by the first two LVs. The coefficients of the 61 selected variables are indicated in Figure 5b, where the variables are reported on the x-axis and the corresponding coefficient is on the y-axis: positive coefficients correspond to variables that show a high first derivative value in 3 h-treated muscle samples, while negative coefficients correspond to variables with a low first derivative value in 3 h-treated muscle samples.

For long-term treatment effects on muscle, instead, the final model contains 314 variables and 1 LV, reaching again the perfect classification of all the samples both in cross-validation and in fitting (Table 1). In this case the first LV explains about 16.6% of the information contained in the X variables and 42.0% of the class membership. The score plot of the first LV is reported in Figure 5c, where the two classes of samples appear well separated along LV_1_. The coefficients of the 314 selected variables are indicated in Figure 5d, where the variables are reported on the x-axis and the corresponding coefficient on the y-axis: positive coefficients correspond to variables with a high first derivative value in 24 h-treated muscle samples, while variables with negative coefficients show a low first derivative value in 24 h-treated muscle samples.

#### 3.3.2. Skin—Short-Term and Long-Term Treatment Effects

**Figure 6:**
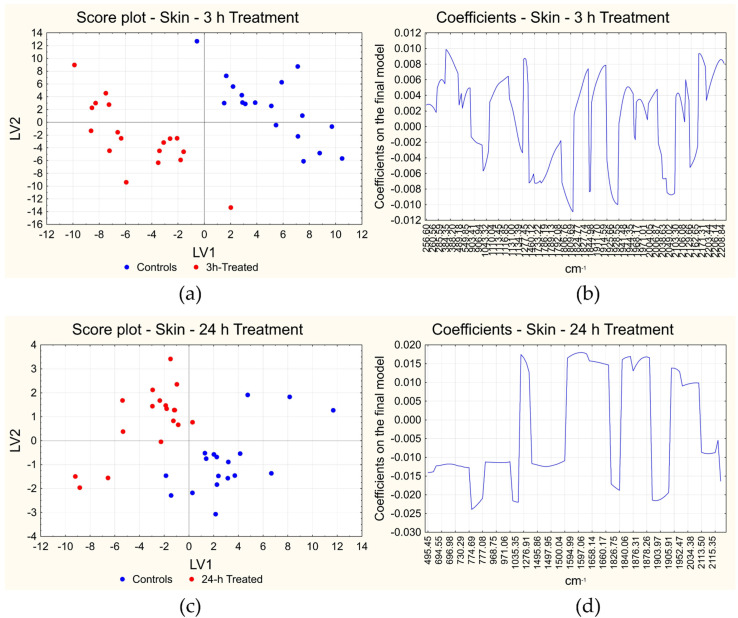
Results of PLS-DA on skin: score plot (**a**) and plot of the coefficients (**b**) of short-term treatment and long-term treatment (**c**,**d**). The score plots report the scores of each measurement in the space given by the first two latent variables (LVs) calculated, with the samples indicated in blue if they are controls and in red if they are treated samples. The plots of the coefficients report the variables included in the final models on the x-axis and the coefficients on the final model on the y-axis. Positive coefficients correspond to variables with a higher signal in the first derivative of treated samples, while negative coefficients correspond to variables with a lower signal in the same situation.

As regards the effects of short-term treatments on skin, the final PLS-DA model contains 273 variables and 2 LVs showing a perfect classification of all the samples both in cross-validation and in fitting (Table 1). In this case, the first two LVs explain about 30.1% of the information contained in the X variables and 45.2% of the class membership. Figure 6a reports the score plot of the first two LVs, where the two classes of samples appear well separated by the first two LVs. The coefficients of the 273 selected variables are instead reported in Figure 6b (variables on the x-axis and the corresponding coefficient on the y-axis): positive coefficients correspond to variables with a high first derivative in 3 h treated skin samples, while negative coefficients correspond to variables with a low first derivative in the same samples.

In the case of long-term effects on skin, the final model contains 108 variables and 3 LVs, reaching again the perfect classification of all the samples in cross-validation and fitting (Table 1). The model with two LVs explains about 39.6% of the information contained in the X variables and the 49.0% of the class membership (Y variable). Figure 6c reports the score plot of the first two LVs, with well separated classes. Figure 6d instead reports the coefficients of the 108 selected variables: variables with positive coefficients show a high first derivative value in 24 h-treated skin samples, while variables with a negative coefficient have a low first derivative value in the same samples.

## 4. Discussion

In the present study, chemometric tools have been coupled to Raman spectroscopy to build models able to discriminate control vs. treated fish samples. PCA was applied as pattern recognition tool to evaluate the presence of groups of samples, in particular related to the investigated matrix (muscle or skin) or to the applied treatment (short- or long-term). PLS-DA was selected as classification tool due to its ability in performing dimensionality reduction since it exploits an approach based on latent variables, thus allowing its application also to datasets where the number of variables is higher than the number of samples (e.g., unlike linear discriminant analysis). Moreover, being a discriminant method, it is devoted to compare different classes: the study was in facts focused on the development of a method for comparing control vs. treated samples in order to identify the differences between them, rather than developing an authentication procedure. In this last case in facts, usually a class of interest is deeply characterized (e.g., by SIMCA method) and then new samples are projected on the built model to verify whether they belong to it.

In the present study, PLS-DA models were able to clearly discriminate controls vs. treated samples for both matrices (muscle and skin) and considering both short-term and long-term treatments; moreover, the built models proved to be reliable and not affected by overfitting as can be argued by the number of variables and latent variables included in the final models.

The analysis of the classification coefficients has allowed the identification of the effect played by the Cafodos-like mixture on muscle and skin. Notwithstanding the fact that the exhaustive interpretation of the effect played by the treatment on the Raman spectrum is beyond the scope of the paper, since a complete interpretation would request the contemporary exploitation of other complementary techniques, some interpretation of the major bands involved in the effect can be attempted. As regards the short-term treatment on muscle samples, the effect is present but has a limited relevance since the number of discriminating variables is low and five LVs are needed to achieve the perfect classification of the samples. Almost all the coefficients of the selected Raman shift values are negative, with the exception of the frequencies in the amide I and III region (between 1290 and 1600 cm^−1^ approximately) [42,62]: this means that the first derivative has particularly low values in these regions or, from another point of view, in the original spectra those bands show a lower slope in treated samples than in control samples. On the other hand, very few regions show a positive coefficient, where the slope of the spectra increases after treatment.

In the case of long-term treatment on muscle, the effect seems more relevant since a higher number of variables has been selected as discriminating and the perfect classification is achieved with just one LV in the final model. There are in particular three spectral regions that show positive coefficients: between 1076 and 1089 cm^−1^, between 1220 and 1230 cm^−1^ and above all between 1659 and 1665 cm^−1^. Based on the previous interpretation of the original Raman spectra on muscle tissue, these regions roughly correspond to the C-C stretching vibration band in β-sheet structures, amide-III and amide-I bands [42]. After treatment, therefore, these bands show a higher slope in the original Raman spectra, i.e., a higher variation. This is probably due to the fact that, thanks to the treatment with chemical reagents, proteins in treated samples maintained their original structure and did not degrade, therefore, they should show stronger signals. Instead, in control samples, the degradation process began, so these bands should change in shape, becoming weaker and broader. This is in agreement with the literature, where it is reported that the spectral components related to the β-sheet structure tend to increase, while the ones related to the α-helix structure decrease during storage, due to denaturation or to the formation of new protein-protein interactions [41,66]. On the other hand, negative coefficients are related to some isolated wavenumbers only (792, 1200, 1416 and 1629 cm^−1^) [47].

In the case of skin, both for short- and long- term treatments, the effects appear more evident than in the case of the short-term treatment on muscle, as it is clear from the higher number of the selected discriminating variables and lower number of LVs included in the final models (Table 1). This behavior is reasonable since the treatment was directly applied on the skin and could have played a more evident effect on muscle only after a long-term treatment. For short-term treatment on skin, in general, the area of the spectrum at lower Raman shift values shows positive coefficients, therefore a higher slope (i.e., a higher variation) in the original spectra. Among the regions showing the highest variation, the signal at ca. 1227 cm^−1^ (part of the amide-III band) [42] can be identified. Moreover, regions at higher Raman shift show a high variation (around 1823–1828 cm^−1^, 1911–1926 cm^−1^, 2170–2210 cm^−1^). Very few regions show instead negative coefficients, i.e., a lower slope after treatment.

Finally, for long-term treatment on skin, high first derivative values can be seen again around 1277 cm^−1^ (amide-III band) [42], and also around the amide-I band (1590–1660 cm^−1^) [42,64,65], similarly to what was encountered with muscle samples. Other regions with positive coefficients (showing a higher variation in the original spectra after treatment) are at 1820–1880 cm^−1^ and 1950–2035 cm^−1^. On the other hand, there are low first derivative values in the regions at low Raman shift values, in particular, between 960 and 1035 cm^−1^ (C-C stretching vibration region) [64,65] and below 800 cm^−1^, including all the area of intermolecular bonds: these areas show a lower slope after treatment in the original spectra. It is probable that on the skin the degradation process is still active to some extent, involving mainly the modification of the tertiary structure of the proteins and maybe the loss of water.

The analysis of the coefficients points out that the effect played by the treatment shows some differences between short- and long-term and, at short-term, is more relevant on skin, while it is active both on muscle and skin at long-term. However, in all the cases, modifications occur that can be identified by micro-Raman spectroscopy, thus, allowing the use of this technique for the rapid screening of fresh fish samples.

## 5. Conclusions

As pointed out in the last FAO report about fishery and aquaculture [1], global consumption of fish and seafood is constantly increasing, at an average annual rate of 3% (thus, exceeding even the population growth rate), together with the emergence of mislabeling, adulteration, misuse of legal and/or illegal additives, incorrect storage practices of fish and other sea products: these frauds for economic purposes often represent a potential hazard for consumers’ health, therefore early in-field detection of this broad range of illicit practices is becoming a common goal of both honest producers and their supply chains.

Spectroscopic techniques, and in particular Raman spectroscopy, coupled to chemometrics, are certainly very promising in the detection of illicit practices and are already widespread for the assessment of food quality and to characterize relevant features of food commodities [50]. Thus, it is likely that spectroscopic techniques will be more and more exploited against food frauds, making available to both consumers and authorities novel tools for reliable food authenticity and safety assessment. In this perspective, the present study focuses on the development of a new and innovative method for uncovering food frauds, which takes advantage of Raman spectroscopy to fill a void left from the current analysis technologies, especially in detection of the effects of compounds such as hydrogen peroxide.

To the best of the authors’ knowledge, this is the first time Raman spectroscopy has been applied for the identification of a Cafodos-like illicit treatment, focusing on both fish muscle and skin evaluation. The procedure could pave the way for a future application directly on the market through the use of a portable device, also considering the rapidity of the spectroscopic measurements when compared to other techniques and the almost absent sample pretreatment required.

In this context, the developed classification models allowed the perfect recognition of all the samples for all the four comparisons carried out, proving that micro-Raman spectroscopy can be applied to identify illicit short- and long-term treatments with Cafodos-like mixtures. Moreover, the application of variable selection procedures allowed us to obtain a simplification of the models, including only the most discriminant variables (i.e., Raman shifts, cm^−1^); this promoted the reduction of overfitting, as also witnessed by the low number of LVs contained in each calculated model.

In addition to the application as a classification tool to assess the presence or not of a particular treatment, the proposed methodology based on Raman spectroscopy coupled to chemometrics could also be applied to monitor food quality in the food industry and perform the assessment of food quality alongside production.

## Figures and Tables

**Table 1:** PLS-DA classification results for the four comparisons investigated: N° of variables and LVs included in each model, %Accuracy (%Acc) and NER%, both in fitting and cross-validation, for the models built on raw data or on first derivative data. Cross-validation was applied with 6 cancellation groups taking out at the same time all the replications of the samples analyzed the same day (1 control and 1 treated fish, 6 total measurements).

		Raw Data				First Derivative			
		N° Variables	N° LV	%Acc	NER%	N° Variables	N° LV	%Acc	NER%
Muscle Short Term	Fitting	38	10	86.11	86.11	61	5	100	100
	Cross-validation			83.33	83.33			100	100
Muscle Long Term	Fitting	558	6	100	100	314	1	100	100
	Cross-validation			100	100			100	100
Skin Short Term	Fitting	470	7	100	100	273	2	100	100
	Cross-validation			100	100			100	100
Skin Long Term	Fitting	294	7	100	100	108	3	100	100
	Cross-validation			100	100			100	100

## Data Availability

Data may be made available upon request to the Corresponding author.
